# Supplementary material for: Quality of Life and Coping Strategies of Palestinian Women with Breast Cancer in the West Bank: A Cross-Sectional Study
Source: Healthcare (Basel). 2025 May 12;13(10):1124. doi: 10.3390/healthcare13101124 (PMC12110817; doi:10.3390/healthcare13101124)
Supplement: Supplementary file 1 [file healthcare-13-01124-s001.zip › healthcare-3594376-supplementary.pdf]

Table S1. QLO Functional Scales and Socio-demographic Variables Associations (N = 147).

Variables	Physical function	Role function	Emotional function	Cognitive function	Social function
	Mean rank	Mean rank	Mean rank	Mean rank	Mean rank
**Place of residence***					
Refugee camp*	84.41	82.00	103.7	81.91	81.82
Village	73.41	71.59	68.33	74.27	72.96
City	72.63	77.03	78.42	71.45	74.23
**Z or X^2**	4.959	1.453	7.544	1.980	0.594
**P-value**	0.695	0.595	**0.022**	0.756	0.797
**Age in years***					
≤ 40	64.49	76.18	90.83	70.21	78.26
41–54	67.51	65.03	74.86	71.82	73.53
≥55	98.23	89.96	53.00	82.81	70.09
**Z or X^2**	8.635	6.386	12.309	1.487	0.350
**P-value**	**0.001**	**0.009**	**0.001**	0.355	0.687
**Marital status****					
Unmarried	80.53	67.82	78.29	84.61	66.71
Married	73.03	74.92	73.36	72.43	75.08
**Z or X^2**	-0.719	-0.727	-0.475	-1.183	-0.825
**P-value**	0.472	0.467	0.635	0.237	0.410
**Education***					
Primary and less	95.10	85.18	60.25	90.45	72.40
Secondary	73.65	74.57	78.04	73.24	78.58
Colleague/University	68.01	70.16	74.49	69.77	70.37
**Z or X^2**	6.704	1.606	1.389	4.339	1.189
**P-value**	**0.043**	0.330	0.261	0.150	0.526
**Working status****					
Employee	59.54	73.70	69.78	65.40	70.17
Housewife	79.59	74.12	75.63	77.33	75.48
**Z or X^2**	-2.573	-0.058	-0.755	-1.548	-0.699
**P-value**	**0.010**	0.954	0.451	0.122	0.484
**Number of children****					
≤5 persons	70.44	73.68	74.08	69.77	72.85
>5 persons	81.59	74.68	73.83	83.00	76.46
**Z or X^2**	-1.488	-0.142	-0.034	-1.786	-0.495
**P-value**	0.137	0.887	0.973	0.074	0.621
**Family size ****					
≤5 persons	72.87	73.62	72.46	68.82	68.85
>5 persons	74.64	74.21	74.87	76.92	76.90
**Z or X^2**	-0.243	-0.086	-0.332	-1.126	-1.136
**P-value**	0.808	0.931	0.740	0.260	0.256
**Monthly salary (US $)***					
< 570	86.65	83.58	77.76	81.84	79.44
570-1140	68.60	68.52	72.11	71.77	73.79
> 1140	62.95	68.27	71.27	65.02	65.23
**Z or X^2**	11.093	7.226	0.829	2.773	5.383
**P-value**	**0.019**	0.084	0.709	0.170	0.304

Significant p-value at $p < 0.05$ (2 tailed). *Variables were tested by Kruskal Wallis test and presented as Chi-square. **Variables were tested by Mann Whitney U test and presented as z value

Table S2. QLO Symptom Scales and Socio-demographic Variables Association (N = 147).

Variables	Fatigue	Nausea/ Vomiting	Pain	Dyspnoea	Insomnia
	Mean rank	Mean rank	Mean rank	Mean rank	Mean rank
**Place of residence***					
Refugee camp*	92.00	78.23	81.36	63.05	85.59
Village	71.19	69.93	70.82	77.63	69.21
City	75.38	81.45	78.81	69.16	81.13
**Z or X^2**	2.566	2.163	2.498	0.954	1.357
**P-value**	0.291	0.234	0.479	0.345	0.177
**Age in years***					
≤ 40	74.98	78.25	78.64	70.24	81.20
41–54	71.65	76.19	67.48	69.67	72.75
≥55	77.73	64.64	82.11	87.21	68.34
**Z or X^2**	0.722	1.024	4.464	4.698	1.373
**P-value**	0.770	0.237	0.165	0.091	0.375
**Marital status****					
Unmarried	76.03	85.97	73.45	79.32	74.08
Married	73.70	72.22	74.08	73.21	73.99
**Z or X^2**	-0.225	-1.477	-0.062	-0.607	-0.009
**P-value**	0.822	0.140	0.951	0.544	0.993
**Education***					
Primary and less	88.18	63.70	90.28	94.08	80.75
Secondary	75.52	72.83	78.24	72.62	76.10
Colleague/University	68.41	78.12	65.34	69.25	70.10
**Z or X^2**	6.627	1.773	5.490	5.281	1.847
**P-value**	0.171	0.312	**0.037**	0.055	0.522
**Working status****					
Employee	63.09	75.98	62.37	69.61	66.39
Housewife	78.22	73.24	78.50	75.70	76.94
**Z or X^2**	-1.956	-0.393	-2.109	-0.810	-1.398
**P-value**	0.051	0.694	**0.035**	0.418	0.162
**Number of children****					
**≤5 persons**	70.35	73.55	73.26	69.16	74.95
>5 persons	81.78	74.96	75.57	84.31	71.99
**Z or X^2**	-1.536	-0.210	-0.315	-2.095	-0.407
**P-value**	0.125	0.834	0.753	**0.036**	0.684
**Family size ****					
≤5 persons	73.99	78.02	75.08	70.75	77.42
>5 persons	74.01	71.73	73.39	75.83	72.07
**Z or X^2**	-0.002	-0.966	-0.235	-0.723	-0.760
**P-value**	0.998	0.334	0.814	0.470	0.447
**Monthly salary (US $)***					
< 570	80.80	75.42	87.48	82.23	85.89
570-1140	69.21	77.00	64.30	71.34	64.70
> 1140	71.66	65.89	69.75	65.19	71.66
**Z or X^2**	1.061	3.038	8.352	2.375	7.442
**P-value**	0.317	0.382	**0.009**	0.140	**0.021**

Significant p-value at $p < 0.05$ (2 tailed). *Variables were tested by Kruskal Wallis test and presented as Chi-square. **Variables were tested by Mann Whitney U test and presented as z value

Table S3. QOL Single-Item Symptom Measures, Global Health Status and Financial Difficulties and Socio-demographic Variables Association (N = 147).

Variables	Appetite Loss	Constipation	Diarrhoea	Financial Difficulties	Global health status
	Mean rank	Mean rank	Mean rank	Mean rank	Mean rank
**Place of residence***					
Refugee camp*	75.09	75.82	67.00	71.91	42.27
Village	68.37	72.39	75.24	77.24	70.55
City	85.50	76.92	73.16	67.74	77.05
**Z or X^2**	4.140	0.902	0.599	2.083	8.997
**P-value**	0.053	0.786	0.765	0.436	**0.033**
**Age in years***					
≤ 40	84.49	67.93	78.98	71.50	70.98
41–54	67.36	80.31	70.40	75.06	76.34
≥55	75.67	67.96	75.73	74.67	72.64
**Z or X^2**	4.313	4.287	1.266	1.126	1.458
**P-value**	0.076	0.124	0.465	0.900	0.792
**Marital status****					
Unmarried	76.03	79.26	71.87	67.87	69.53
Married	73.70	73.22	74.32	74.91	74.66
**Z or X^2**	-0.246	-0.669	-0.274	-0.705	-0.497
**P-value**	0.806	0.503	0.784	0.481	0.619
**Education***					
Primary and less	73.48	69.55	83.63	84.00	53.63
Secondary	75.93	71.37	67.73	77.07	69.10
Colleague/University	72.43	77.69	76.74	68.27	84.47
**Z or X^2**	0.049	1.882	2.769	3.580	12.886
**P-value**	0.875	0.528	0.169	0.237	**0.008**
**Working status****					
Employee	71.59	68.70	79.17	65.39	83.80
Housewife	74.93	76.05	72.00	77.33	70.21
**Z or X^2**	-0.472	-1.089	-1.071	-1.597	-1.757
**P-value**	0.637	0.276	0.284	0.110	0.079
**Number of children****					
**≤5 persons**	73.49	72.79	79.17	70.47	73.07
>5 persons	75.10	76.59	72.00	81.52	75.99
**Z or X^2**	-0.236	-0.585	-0.265	-1.538	-0.393
**P-value**	0.813	0.559	0.791	0.124	0.694
**Family size ****					
≤5 persons	79.51	69.37	76.16	72.66	75.72
>5 persons	70.89	76.61	72.78	74.76	73.03
**Z or X^2**	-1.301	-1.148	-0.540	-0.300	-0.372
**P-value**	0.193	0.251	0.589	0.764	0.710
**Monthly salary (US $)***					
< 570	77.69	77.49	80.95	85.08	64.64
570-1140	71.74	67.74	68.33	69.42	78.22
> 1140	72.09	80.05	73.08	64.03	81.75
**Z or X^2**	0.684	1.827	2.822	10.677	3.364
**P-value**	0.676	0.209	0.176	**0.035**	0.112

Significant p-value at $p < 0.05$ (2 tailed). *Variables were tested by Kruskal Wallis test and presented as Chi-square. **Variables were tested by Mann Whitney U test and presented as z value

Table S4. Coping and socio-demographic Variables Association (N = 147).

Variables	Coping	Positive Focus	Diversion	Planning	Individual scale	Interpersonal scale
	Mean rank	Mean rank	Mean rank	Mean rank	Mean rank	Mean rank
**Place of residence***						
Refugee camp*	63.50	62.23	41.95	58.73	56.82	69.64
Village	68.97	70.97	75.57	70.83	70.83	70.58
City	87.15	83.27	78.73	84.45	84.92	82.24
**Z or X^2**	4.491	3.833	2.400	3.566	3.765	2.304
**P-value**	**0.045**	0.177	**0.029**	0.093	0.074	0.293
**Age in years***						
≤ 40	87.51	76.14	74.84	80.39	80.75	79.13
41–54	75.60	79.04	73.83	77.22	77.33	77.81
≥55	55.27	61.19	73.40	60.07	59.44	60.30
**Z or X^2**	4.948	1.573	0.077	3.515	0.771	1.476
**P-value**	**0.004**	0.112	0.988	0.072	0.062	0.083
**Marital status****						
Unmarried	65.42	53.66	61.71	58.34	57.95	27.68
Married	75.27	77.02	75.82	76.32	76.38	80.88
**Z or X^2**	-0.946	-2.255	-1.364	-1.751	-1.76	-5.189
**P-value**	0.344	**0.024**	0.173	0.080	0.078	**0.000**
**Education***						
Primary and less	36.93	40.23	47.85	32.03	35.38	55.38
Secondary	70.60	69.11	70.61	67.73	69.60	69.91
Colleague/University	88.11	88.46	84.84	92.15	89.47	83.22
**Z or X^2**	11.46	17.28	14.37	27.78	19.86	4.507
**P-value**	**0.000**	**0.000**	**0.001**	**0.000**	**0.000**	**0.001**
**Working status****						
Employee	80.07	86.45	80.22	89.52	84.67	80.34
Housewife	71.65	69.18	71.59	68.00	69.87	71.55
**Z or X^2**	-1.081	-2.228	-1.114	-2.803	-1.892	-1.147
**P-value**	0.280	**0.026**	0.265	0.005	0.059	0.251
**Number of children****						
**≤5 persons**	77.59	75.13	76.61	77.39	77.08	74.44
>5 persons	66.37	71.60	68.46	66.79	67.45	73.07
**Z or X^2**	-1.496	-0.474	-1.094	-1.436	-1.28	-0.185
**P-value**	0.135	0.635	0.274	0.151	0.200	0.854
**Family size ****						
≤5 persons	70.22	70.47	68.03	70.56	69.46	65.66
>5 persons	76.13	75.99	77.37	75.94	76.56	78.70
**Z or X^2**	-0.813	-0.762	-1.292	-.751	-0.97	-1.821
**P-value**	0.416	0.446	0.197	0.453	0.331	0.069
**Monthly salary (US $)***						
< 570	72.41	68.88	73.95	65.36	70.27	63.26
570-1140	74.61	72.81	73.86	78.57	74.08	78.87
> 1140	75.53	84.91	74.34	79.86	80.14	82.84
**Z or X^2**	0.514	2.929	0.135	2.220	1.692	3.213
**P-value**	0.937	0.224	0.999	0.159	0.582	0.054

Significant p-value at $p < 0.05$ (2 tailed). *Variables were tested by Kruskal Wallis test and presented as Chi-square. **Variables were tested by Mann Whitney U test and presented as z value

Table S5. QOL Functional Scales and Clinical Characteristics and Social Support Association (N = 147).

Variables	Physical function	Role function	Emotional function	Cognitive function	Social function
	Mean rank	Mean rank	Mean rank	Mean rank	Mean rank
**Diagnosis duration (months)***					
Less than 3	73.09	73.88	93.20	81.12	78.91
3– 6	73.14	73.36	76.72	73.21	68.21
6-12	75.81	74.91	55.34	69.36	77.47
**Z or X^2**	1.240	0.182	17.798	3.434	2.405
**P-value**	0.937	0.980	**0.000**	0.426	0.354
**Stage at diagnosis***					
Stage 1	58.64	80.82	76.41	75.45	76.36
Stage 2	73.67	75.70	81.42	74.99	76.44
Stage 3	73.96	71.64	71.22	74.10	71.72
Stage 4	91.32	72.59	49.36	66.68	72.64
**Z or X^2**	5.457	1.556	4.740	0.288	1.732
**P-value**	0.350	0.878	0.118	0.944	0.929
**Chemotherapy****					
Yes	74.20	73.12	72.84	73.52	73.56
NO	68.30	99.10	106.90	87.60	86.50
**Z or X^2**	-0.306	-1.437	-1.775	-.739	-0.689
**P-value**	0.760	0.151	0.076	0.460	0.491
**Radiotherapy****					
Yes	73.49	72.20	64.69	65.84	71.23
NO	74.22	74.77	77.98	77.49	75.18
**Z or X^2**	-0.096	-0.358	-1.749	-1.544	-0.532
**P-value**	0.924	0.720	0.080	0.123	0.595
**Hormonal therapy****					
Yes	67.75	79.54	57.00	56.71	71.79
NO	74.66	73.42	75.79	75.82	74.23
**Z or X^2**	-0.580	-0.548	-1.586	-1.624	-0.211
**P-value**	0.562	0.584	0.113	0.104	0.833
**Biological therapy****					
Yes	59.91	74.27	72.34	69.66	66.00
NO	77.32	73.94	74.39	75.02	75.88
**Z or X^2**	-1.955	-0.040	-.232	-0.609	-1.140
**P-value**	0.051	0.968	0.817	0.542	0.254
**Surgical treatment****					
Yes	74.85	75.30	65.95	71.72	71.34
NO	72.77	72.12	85.68	77.31	77.86
**Z or X^2**	-0.293	-0.477	-2.788	-0.795	-0.941
**P-value**	0.770	0.633	**0.005**	0.426	0.347
**Surgical intervention***					
Total mastectomy	76.23	75.18	64.98	72.07	72.43
Partial mastectomy	69.92	75.71	69.42	70.47	67.42
No surgical intervention	72.77	72.12	85.68	77.31	77.86
**Z or X^2**	0.095	0.980	5.374	4.238	1.274
**P-value**	0.813	0.891	**0.019**	0.721	0.576
**Chronic diseases****					
Yes	85.87	80.52	68.51	79.15	79.69

NO	68.07	70.74	76.74	71.42	71.15
**Z or X²**	-2.400	-1.407	-1.116	-1.055	-1.182
**P-value**	**0.016**	0.160	0.264	0.291	0.237
**Taking pain medication****					
Yes	79.56	77.13	78.28	76.56	75.36
NO	65.71	69.33	67.62	70.19	71.97
**Z or X²**	-1.941	-1.166	-1.502	-0.904	-0.489
**P-value**	0.052	0.243	0.133	0.366	0.625
**History of relative cancer****					
Yes	76.72	75.21	76.31	77.72	78.64
NO	71.17	72.74	71.59	70.13	69.17
Z or X²	-0.794	-.0376	-.679	-1.099	-1.391
**P-value**	0.427	0.707	0.497	0.272	0.164
**Type of relative cancer***					
Breast cancer	72.42	75.88	83.70	77.65	82.48
Other cancers	83.98	72.84	66.39	76.44	73.48
No cancer history	70.51	73.39	71.59	70.74	69.17
**Z or X²**	2.645	0.693	5.367	2.117	3.814
**P-value**	0.313	0.932	0.169	0.646	0.246
**Family support****					
Yes	75.24	74.26	73.32	73.48	73.72
NO	67.33	72.59	77.67	76.83	75.50
**Z or X²**	-0.822	-0.186	-.455	-0.352	-0.190
**P-value**	0.411	0.853	0.649	0.725	0.850
**Husband support****					
Yes	61.17	74.82	85.21	68.24	82.17
NO	80.22	73.60	68.57	76.79	70.04
**Z or X²**	-2.556	-0.175	-2.244	-1.161	-1.670
**P-value**	**0.011**	0.861	**0.025**	0.246	0.095
**Other sources of support****					
Yes	73.68	78.25	69.07	77.21	70.43
NO	74.08	73.00	75.16	73.24	74.84
**Z or X²**	-0.045	-0.629	-0.687	-0.451	-0.509
**P-value**	0.964	0.529	0.492	0.652	0.611

Significant p-value at $p < 0.05$ (2 tailed). *Variables were tested by Kruskal Wallis test and presented as Chi-square. **Variables were tested by Mann Whitney U test and presented as z value

Table S6. QOL Symptom Scales and Clinical Characteristics and Social Support Association (N = 147).

**Variables**	**Fatigue**	**Nausea/ Vomiting**	**Pain**	**Dyspnoea**	**Insomnia**
	Mean rank	Mean rank	Mean rank	Mean rank	Mean rank
**Diagnosis duration (months)***					
Less than 3	83.22	81.93	78.70	78.49	81.38
3– 6	73.75	77.57	72.70	70.54	73.95
6-12	67.02	63.18	71.93	74.84	68.22
**Z or X^2**	1.961	6.536	2.821	0.223	3.419
**P-value**	0.207	**0.047**	0.718	0.633	0.336
**Stage at diagnosis***					
Stage 1	75.64	85.68	71.41	70.36	86.68
Stage 2	76.30	75.29	74.91	75.26	72.29
Stage 3	71.10	70.21	71.46	75.95	73.62
Stage 4	77.91	78.59	87.23	59.14	72.64
**Z or X^2**	0.203	3.975	2.125	1.746	0.003
**P-value**	0.896	0.584	0.698	0.626	0.761
**Chemotherapy****					
Yes	72.88	73.81	72.46	73.57	73.38
NO	105.90	79.50	117.80	86.10	91.70
**Z or X^2**	-1.725	-.330	-2.396	-0.673	-0.981
**P-value**	0.085	.741	**0.017**	0.501	0.326
**Radiotherapy****					
Yes	69.11	65.76	73.15	68.43	70.39
NO	76.09	77.52	74.36	76.38	75.54
**Z or X^2**	-0.920	-1.724	-0.162	-1.079	-0.698
**P-value**	0.358	0.085	0.871	0.281	0.485
**Hormonal therapy****					
Yes	57.57	67.39	72.18	87.39	65.75
NO	75.73	74.70	74.19	72.59	74.87
**Z or X^2**	-1.536	-0.686	-0.172	-1.288	-0.791
**P-value**	0.125	0.493	0.863	0.198	0.429
**Biological therapy****					
Yes	68.52	68.32	72.71	65.66	66.93
NO	75.29	75.34	74.30	75.96	75.66
**Z or X^2**	-0.766	-0.882	-0.182	-1.199	-1.013
**P-value**	0.444	0.378	0.856	0.230	0.311
**Surgical treatment****					
Yes	71.83	66.92	74.67	75.37	68.12
NO	77.14	84.27	73.03	72.02	82.53
**Z or X^2**	-0.752	-2.729	-0.234	-0.488	-2.091
**P-value**	0.452	**0.006**	0.815	0.625	**0.036**
**Surgical intervention***					
Total mastectomy	69.60	68.22	74.56	76.91	67.85
Partial mastectomy	79.84	62.26	75.05	69.84	69.11
No surgical intervention	77.14	84.27	73.03	72.02	82.53
**Z or X^2**	3.828	6.344	0.565	1.884	1.912
**P-value**	0.485	**0.020**	0.972	0.711	0.111
**Chronic diseases****					
Yes	81.26	73.15	74.38	80.11	68.34

NO	70.37	74.42	73.81	70.94	76.83
**Z or X²**	-1.478	-0.192	-0.078	-1.281	-1.183
**P-value**	0.139	0.848	0.938	0.200	0.237
**Taking pain medication****					
Yes	82.02	74.43	83.09	80.22	79.80
NO	62.04	73.36	60.44	64.73	65.36
**Z or X²**	-2.821	-0.169	-3.237	-2.251	-2.091
**P-value**	**0.005**	0.866	**0.001**	**0.024**	**0.037**
**History of relative cancer****					
Yes	75.01	75.69	76.36	74.43	72.18
NO	72.94	72.24	71.54	73.56	75.90
**Z or X²**	-0.298	-0.553	-0.702	-0.129	-0.549
**P-value**	0.766	0.580	0.483	0.897	0.583
**Type of relative cancer***					
Breast cancer	79.56	77.88	81.74	70.15	76.62
Other cancers	69.73	74.77	70.25	82.64	68.61
No cancer history	72.58	71.34	71.04	72.46	74.83
**Z or X²**	2.366	1.438	4.793	2.310	2.721
**P-value**	0.560	0.664	0.347	0.384	0.685
**Family support****					
Yes	75.06	73.70	74.65	73.72	75.81
NO	68.26	75.61	70.52	75.50	64.22
**Z or X²**	-0.712	-0.222	-0.437	-0.192	-1.245
**P-value**	0.476	0.824	0.662	0.848	0.213
**Husband support****					
Yes	78.28	80.88	73.38	71.21	74.03
NO	71.92	70.67	74.30	75.35	73.98
**Z or X²**	-0.859	-1.532	-0.127	-0.576	-0.006
**P-value**	0.390	0.125	0.899	0.564	0.995
**Other sources of support****					
Yes	69.29	81.52	70.61	62.89	67.95
NO	75.11	72.23	74.80	76.61	75.42
**Z or X²**	-0.659	-1.167	-0.480	-1.597	-0.867
**P-value**	0.510	0.243	0.631	0.110	0.386

Significant p-value at $p < 0.05$ (2 tailed). *Variables were tested by Kruskal Wallis test and presented as Chi-square. **Variables were tested by Mann Whitney U test and presented as z value

Table S7. QOL Single-Items, Global Health Status, Financial Difficulties and Clinical Characteristics and Social Support Association (N = 147).

Variables	Appetite Loss	Constipation	Diarrhoea	Financial Difficulties	Global health status
	Mean rank	Mean rank	Mean rank	Mean rank	Mean rank
**Diagnosis duration (months)***					
Less than 3	90.63	63.74	71.87	71.05	72.05
3– 6	75.10	83.47	75.82	70.45	79.65
6-12	59.44	70.09	73.38	80.84	68.36
**Z or X^2**	16.283	7.007	0.625	1.076	2.606
**P-value**	**0.001**	**0.023**	0.862	0.363	0.360
**Stage at diagnosis***					
Stage 1	103.14	58.18	73.68	87.27	90.05
Stage 2	72.09	80.73	73.20	72.98	77.41
Stage 3	68.72	71.93	73.62	71.95	68.20
Stage 4	87.14	66.91	80.86	78.59	75.32
**Z or X^2**	8.652	4.561	0.486	2.488	4.351
**P-value**	**0.030**	0.203	0.935	0.678	0.353
**Chemotherapy****					
Yes	72.91	73.76	74.38	74.07	74.30
NO	105.00	80.80	63.30	72.10	65.50
**Z or X^2**	-1.830	-0.421	-0.669	-0.106	-0.460
**P-value**	0.067	0.674	0.504	0.915	0.646
**Radiotherapy****					
Yes	65.14	74.00	73.55	76.26	70.55
NO	77.79	74.00	74.19	73.03	75.48
**Z or X^2**	-1.822	0.000	-0.099	-0.441	-0.651
**P-value**	0.068	1.000	0.921	0.659	0.515
**Hormonal therapy****					
Yes	61.43	77.11	66.86	71.36	81.50
NO	75.32	73.67	74.75	74.28	73.21
**Z or X^2**	-1.283	-0.333	-0.772	-0.256	-0.701
**P-value**	0.199	0.739	0.440	0.798	0.483
**Biological therapy****					
Yes	66.14	70.98	71.09	68.73	71.13
NO	75.85	74.71	74.68	75.24	74.68
**Z or X^2**	-1.199	-0.483	-0.470	-0.762	-0.402
**P-value**	0.231	0.629	0.638	0.446	0.688
**Surgical treatment****					
Yes	66.44	77.53	75.08	75.98	72.45
NO	84.96	68.88	72.43	71.13	76.25
**Z or X^2**	-2.863	-1.402	-0.433	-0.712	-0.538
**P-value**	**0.004**	0.161	0.665	0.476	0.590
**Surgical intervention***					
Total mastectomy	65.08	79.57	76.48	76.41	70.95
Partial mastectomy	71.32	70.24	70.08	74.45	77.82
No surgical intervention	84.96	68.88	72.43	71.13	76.25
**Z or X^2**	9.851	0.232	0.751	0.142	0.976
**P-value**	**0.014**	0.078	0.724	0.763	0.710

**Chronic diseases****					
Yes	71.29	72.78	75.72	79.12	70.21
NO	75.36	74.61	73.14	71.44	75.89
**Z or X^2**	-0.604	-0.286	-0.406	-1.080	-0.771
**P-value**	0.546	0.775	0.685	0.280	0.440
**Taking pain medication****					
Yes	79.64	77.57	75.09	75.88	69.03
NO	65.59	68.67	72.38	71.19	81.42
**Z or X^2**	-2.165	-1.441	-0.441	-.685	-1.750
**P-value**	**0.030**	0.150	0.659	0.493	0.080
**History of relative cancer****					
Yes	75.29	74.09	74.90	77.34	77.80
NO	72.66	73.90	73.06	70.52	70.04
**Z or X^2**	-0.413	-0.031	-0.306	-1.017	-1.118
**P-value**	0.680	0.975	0.760	0.309	0.264
**Type of relative cancer***					
Breast cancer	80.37	72.93	77.30	75.79	75.87
Other cancers	71.20	75.66	71.67	79.42	80.98
No cancer history	71.44	73.90	73.06	70.52	69.78
**Z or X^2**	1.586	0.194	0.546	1.777	0.864
**P-value**	0.436	0.950	0.766	0.554	0.429
**Family support****					
Yes	73.83	74.77	75.64	74.57	71.40
NO	74.89	69.83	65.17	70.91	88.02
**Z or X^2**	-0.121	-0.593	-1.266	-0.397	-1.740
**P-value**	0.904	0.553	0.205	0.692	0.082
**Husband support****					
Yes	76.84	72.39	66.91	76.93	77.38
NO	72.62	74.78	77.44	72.58	72.36
**Z or X^2**	-0.623	-0.371	-1.645	-0.608	-0.677
**P-value**	0.533	0.711	0.100	0.543	0.498
**Other sources of support****					
Yes	72.93	73.75	73.11	77.93	87.39
NO	74.25	74.06	74.21	73.08	70.85
**Z or X^2**	-0.163	-0.040	-0.144	-0.568	-1.872
**P-value**	0.870	0.968	0.885	0.570	0.061

Significant p-value at $p < 0.05$ (2 tailed). *Variables were tested by Kruskal Wallis test and presented as Chi-square. **Variables were tested by Mann Whitney U test and presented as z value

Table S8. Coping and Clinical Characteristics and Social Support Association (N = 147).

Variables	Coping	Positive Focus	Diversion	Planning	Individual scale	Interpersonal scale
	Mean rank	Mean rank	Mean rank	Mean rank	Mean rank	Mean rank
**Diagnosis duration (months)***						
Less than 3	77.00	73.55	71.59	71.30	72.37	74.58
3– 6	81.45	80.47	81.26	79.66	81.29	76.65
6-12	62.16	66.14	66.68	68.95	66.03	70.18
**Z or X²**	3.637	5.185	5.869	2.510	1.534	1.118
**P-value**	0.054	0.211	0.183	0.372	0.171	0.720
**Stage at diagnosis***						
Stage 1	3.637	95.95	84.05	100.55	95.77	97.82
Stage 2	0.054	74.26	69.65	71.49	70.83	73.22
Stage 3	3.637	71.40	76.13	72.19	73.92	71.12
Stage 4	0.054	66.55	73.95	71.73	69.45	71.82
**Z or X²**	4.044	3.461	0.259	1.903	0.670	2.377
**P-value**	0.341	0.310	0.701	0.186	0.343	0.266
**Chemotherapy****						
Yes	73.22	73.60	73.65	73.55	73.46	73.62
NO	96.10	85.50	84.00	86.80	89.30	84.80
**Z or X²**	-1.187	-0.621	-0.540	-0.697	-0.818	-0.589
**P-value**	0.235	0.535	0.589	0.486	0.413	0.556
**Radiotherapy****						
Yes	66.70	66.73	72.97	68.06	68.32	74.66
NO	77.12	77.11	74.44	76.54	76.43	73.72
**Z or X²**	-1.365	-1.368	-0.195	-1.128	-1.059	-0.125
**P-value**	0.172	0.171	0.846	0.259	0.290	0.900
**Hormonal therapy****						
Yes	83.57	80.32	83.50	82.43	83.61	75.00
NO	72.99	73.33	73.00	73.11	72.99	73.89
**Z or X²**	-.889	-0.590	-0.888	-0.794	0-.889	-0.094
**P-value**	0.374	0.555	0.375	0.427	0.374	0.925
**Biological therapy****						
Yes	79.84	77.13	76.46	77.75	78.80	69.54
NO	72.63	73.26	73.42	73.12	72.87	75.05
**Z or X²**	-0.811	-0.436	-0.344	-0.528	-0.66	-0.630
**P-value**	0.418	0.663	0.731	0.597	0.507	0.529
**Surgical treatment****						
Yes	68.34	70.71	73.44	71.13	70.07	71.31
NO	82.21	78.77	74.81	78.16	79.70	77.90
**Z or X²**	-1.951	-1.139	-0.193	-1.003	-1.349	-0.942
**P-value**	0.051	0.255	0.847	0.316	0.177	0.346
**Surgical intervention***						
Total mastectomy	70.79	70.90	74.85	73.43	71.71	70.55
Partial mastectomy	59.55	70.05	68.42	62.92	64.21	74.03
No surgical intervention	82.21	78.77	74.81	78.16	79.70	77.90
**Z or X²**	5.023	5.533	1.435	2.691	5.041	1.143
**P-value**	0.088	0.521	0.826	0.378	0.319	0.609
**Chronic diseases****						
Yes	62.35	65.15	71.26	61.88	64.30	65.54

NO	79.83	78.42	75.37	80.06	78.85	78.23
**Z or X²**	-2.358	-1.800	-0.559	-2.48	-1.956	-1.740
**P-value**	**0.018**	0.072	0.576	**0.013**	0.050	0.082
**Taking pain medication****						
Yes	72.43	70.88	69.47	71.11	70.53	70.70
NO	76.34	78.65	80.76	78.31	79.17	78.92
**Z or X²**	-0.548	-1.096	-1.595	-1.023	-1.207	-1.172
**P-value**	0.584	0.273	0.111	0.306	0.228	0.241
**History of relative cancer****						
Yes	76.44	77.04	78.83	73.50	76.32	77.75
NO	71.46	70.83	68.97	74.52	71.58	70.09
**Z or X²**	-0.713	-0.893	-1.421	-.148	-.675	-1.114
**P-value**	0.476	0.372	0.155	0.882	0.500	0.265
**Type of relative cancer***					76.32	
Breast cancer	78.69	80.43	81.10	73.71	78.85	80.17
Other cancers	75.25	75.88	79.27	75.81	75.78	74.50
No cancer history	70.65	69.33	67.42	73.37	70.31	70.09
**Z or X²**	0.665	3.458	1.075	1.102	0.611	6.324
**P-value**	0.605	0.377	0.175	0.961	0.561	0.454
**Family support****						
Yes	71.88	72.04	70.58	72.68	71.67	73.25
NO	85.41	84.57	92.46	81.13	86.54	78.02
**Z or X²**	-1.407	-1.309	-2.289	-0.89	-1.540	-0.504
**P-value**	0.160	0.190	**0.022**	0.373	0.124	0.614
**Husband support****						
Yes	85.45	86.33	78.32	86.24	86.06	99.66
NO	68.45	68.02	71.90	68.07	68.15	61.56
**Z or X²**	-2.281	-2.471	-0.867	-2.474	-2.394	-5.195
**P-value**	**0.023**	**0.013**	0.386	**0.013**	**0.017**	**0.000**
**Other sources of support****						
Yes	81.66	86.46	88.09	84.71	87.16	76.61
NO	72.20	71.07	70.68	71.48	70.90	73.39
**Z or X²**	-1.063	-1.740	-1.968	-1.509	-1.820	-0.368
**P-value**	0.288	0.082	**0.050**	0.131	0.069	0.713

Significant p-value at $p < 0.05$ (2 tailed). *Variables were tested by Kruskal Wallis test and presented as Chi-square. **Variables were tested by Mann Whitney U test and presented as z value
